# Effect of magnification devices on dental students’ visual acuity

**DOI:** 10.1371/journal.pone.0212793

**Published:** 2019-03-27

**Authors:** Danielle Wajngarten, Patrícia Petromilli Nordi Sasso Garcia

**Affiliations:** Department of Social Dentistry, São Paulo State University (Unesp), School of Dentistry, Araraquara, Araraquara, São Paulo, Brazil; University of Ontario Institute of Technology, CANADA

## Abstract

This study aimed to determine dental students’ visual acuity and neck angulation when using magnification devices and distances from the operating field. Forty students from each of the second through fifth years of the five-year program at the School of Dentistry of Araraquara were selected (N = 160). Visual acuity was tested using a miniature Snellen eye chart under five different settings (naked eye; simple loupe; Galilean loupe; Keplerian loupe and an operating microscope). Photographs were taken during the visual acuity exam in order to evaluate the angulation of the subjects’ necks in a neutral posture. The two-factor analysis of variance and the Games-Howell post-hoc test were performed (α = 0.05). A significant difference in visual acuity and neck angulation was found between the "magnification device" and "distance" factors in each of the graduating classes analyzed (p<0.05). At a standardized distance, the Keplerian loupe (535.93±133.69), the Galilean loupe (514.06±171.56), and the operating microscope (517.71±161.61) all provided greater visual acuity. At a subjectively comfortable distance, the Keplerian (521.35±157.99) and Galilean (515.00±156.32) loupes produced the best visual acuity. The angulation of the neck was greater when the simple loupes (56,59±10,88) and naked eye (56.51±13.55) were used at a subjectively comfortable distance. At both a standardized distance and a comfortable distance, the Galilean and Keplerian magnification systems provided the best visual acuity and the lowest angulation of the operator’s neck. At a standardized distance of 30 cm to 40 cm, the operating microscopes produced similar results.

## Introduction

While performing surgical procedures, dental surgeons may incline and/or twist their head, neck, and torso [1,2] to get closer to the patient’s mouth [2] and to obtain a better view of the operating field. This strategy has caused many of these professionals to fail to maintain a neutral posture [3], to strengthen the muscles involved on one side of the body, and to weaken and stretch the muscles on the other side of the body. Muscle shortening causes asymmetrical spine strength and muscle ischemia [4], thus increasing professionals’ risk of developing musculoskeletal disorders [1,5,6].

According to Forgie et al [7], Meraner and Nase [8] and Congdon et al [9], this risk to dental surgeons’ postures can be lowered with the use of magnifying devices. These devices improve visualization of the operating field [10,11] by magnifying oral structures and, in doing so, improve the positioning of the neck and back muscles [12] and thus decrease the surgeon’s risk of developing musculoskeletal disorders [13,14]. Improving surgeons’ visual acuity during procedures is therefore important both for their ability to properly and precisely perform said procedures [11] and for their occupational health [1]. Furthermore, the use of magnification presupposes the need to maintain a set focal range, and thus limits the surgeon’s extension of his or her neck [15].

Magnification devices have been used by professionals in a variety of fields and have been implemented in dentistry as well [16]. These devices vary from simple loupes, which have lower magnification power, to operating microscopes, which may provide up to 30x magnification [10,17].

There are three fundamental types of magnifying loupes. They are classified based on the method through which magnification is produced: simple loupes, the Galilean loupe, and the Keplerian loupe [18]. These devices differ in the types and positions of the lenses used in their structures. According to Perrin et al [19], simple loupes consist of a pair of positive meniscus lenses positioned side by side. Loupes in the Galilean system are conical, and their optic system consists of concave and convex lenses. Loupes in the Keplerian system are cylindrical in shape and are longer due to their complex internal system of convex lenses and prisms.

Though magnification has long been recommended for improving dentistry procedures, its routine use is relatively recent [1,7,12]. Few studies have presented scientific evidence on the extent of improvement to visual acuity provided by different magnifying systems and the quality of dental surgeons’ postures [1,12].

Because these devices can improve dentistry professionals’ visual acuity and occupational health even while they are still completing their studies, this study aimed to determine dentistry students’ visual acuity and neck angulation during their use of different magnification devices and distances from the operating field. The null hypothesis tested was that the different magnification systems used (simple loupes, the Galilean system, the Keplerian system, and an operating microscope) would not affect neck angulation or visual acuity.

## Materials and methods

### Study design and sample selection

The subjects in this study were undergraduate students in the dentistry program at the School of Dentistry of the Araraquara campus of São Paulo State University (UNESP) who provided a written informed consent to participate. The exclusion criterion was student age; students 40 years of age or older were excluded. The age cut-off of 40 years was chosen because it is at this time that natural changes in the eye begin [20].

Forty students from each of the second through fifth years of the five-year program were selected (N = 160). The sample size was calculated during a pilot study and was based on the means and standard deviations of the experimental groups, with a power of 80% and a significance level of 5%.

The dependent variables were each subject’s visual acuity and angulation of the neck. These variables were evaluated as each subject used the magnification devices. The independent variables were the magnification devices in five different settings (under the naked eye; using a simple loupe with 3.5x magnification [Bioart]; using a Galilean dental loupe with 3.5x magnification [Ymarda Optical Instrument Factory]; using a Keplerian loupe with 4.0x magnification [Ymarda Optical Instrument Factory]; and using an operating microscope with 6.0x magnification [DF Vasconcelos]) and the working distance between the operator’s eyes and the dental mannequin’s mouth under two different conditions (recommended distance of approximately 35 cm [21] or at a subjectively comfortable distance chosen by the operator).

This study was submitted to and approved by the Research Ethics Committee of the School of Dentistry of São Paulo State University (UNESP), Araraquara (CAAE Registry No. 54753816.9.0000.5416).

### Measuring visual acuity

Visual acuity was tested using a miniature Snellen eye chart, which allowed for the test to be performed at a distance typical to that between dental surgeons and their operating fields [11]. The Es on the eye chart ranged from 0.01 mm to 0.04 mm in size [22].

**Fig 1:**
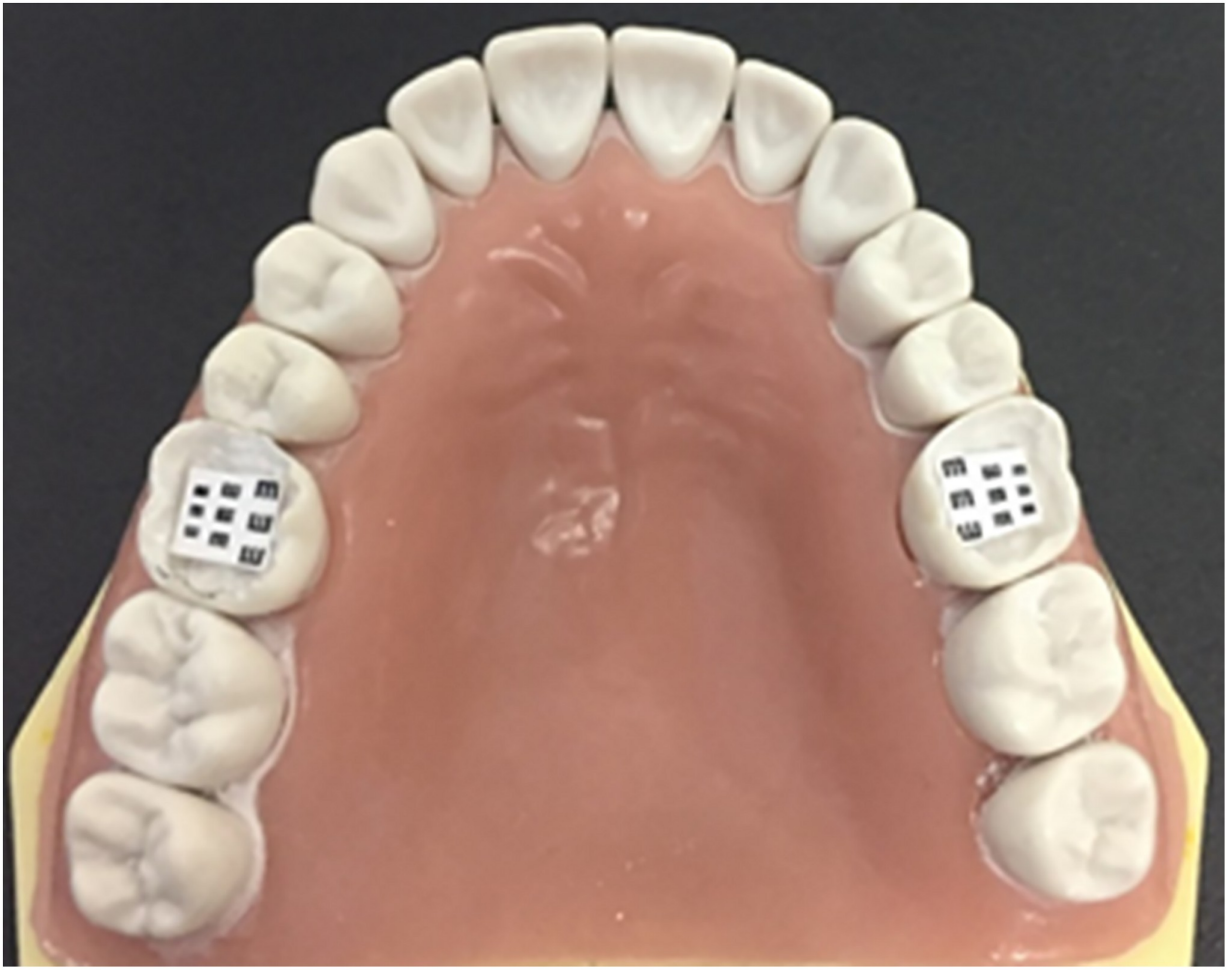
Miniature Snellen charts placed on maxillary first molars.

The tests were performed on the maxillary first molars of a dental mannequin (MOM, Marília, São Paulo). Each student’s dominant side was considered—right-handed students worked on the right maxillary first molar, while left-handed students worked on the left maxillary first molar. The dominant hand was the one which students used primarily to work and was reported by each student before the tests. Cavities were created in the occlusal surfaces of these teeth at depths similar to those of a class I cavity preparation (Fig 1).

The last line on the chart that subjects were able to read correctly was selected to calculate their visual acuity [22,23]. Visual acuity was calculated using the equation VA = 1/AVA, where VA represented visual acuity and AVA represented angular visual acuity.

Angular visual acuity is defined as the smallest angle (in minutes) at which two points can be recognized separately. In vision exams, these points are the spaces between the bars of the letter E (one fifth of the total size of the letter E). To calculate angular visual acuity, the Epsilon angle must first be calculated using the following equation:

$$\varepsilon ={tan}^{-1}\left(\frac{d}{a}\right)$$

where

ε = the Epsilon angle,

d = the space between the bars of the letter E, and

a = the distance between the surgeon’s eyes and the patient’s teeth.

After obtaining the Epsilon angle, angular visual acuity can be calculated using the equation $AVA=\frac{1}{\left(\varepsilon x60\right)}$.

In this study, visual acuity has been expressed as cycles per degree (C/°), which is the conventional unit of measurement when square Snellen charts are used [24].

### Procedures for evaluating visual acuity

The dental mannequin containing the tooth with the miniature Snellen chart was placed in a dental chair to simulate clinical conditions. A different version of the miniature Snellen chart was used in each magnification device test. The charts used were chosen at random and varied in order to keep subjects from memorizing the tests.

Each test was performed with the subject seated in the dentist’s chair in such a way that his or her thighs were parallel to the floor and a 90-degree angle was formed between his or her legs. The back of the dental chair was positioned at an angle of approximately 180 degrees, and care was taken to keep the chair from pressing on the subject’s legs during the evaluation. The dental mannequin’s head was laid back, and the dental light was placed above and opposite the mouth of the mannequin, since the Snellen chart had been placed on an upper tooth.

The visual acuity exam was performed using the direct view of the tooth, without the aid of mouth mirrors. During the visual acuity tests, the distance between the operator’s eyes and the mannequin’s tooth was measured using measuring tape. These measurements were written down on visual acuity test cards. The dental chair, mannequin, and amount of lighting were consistent across all of the tests performed in this study. Additionally, lens adjustments, eye–typodont distance, and the reading of the E-optotypes were all controlled by the same researcher.

### Evaluating angulation

**Fig 2:**
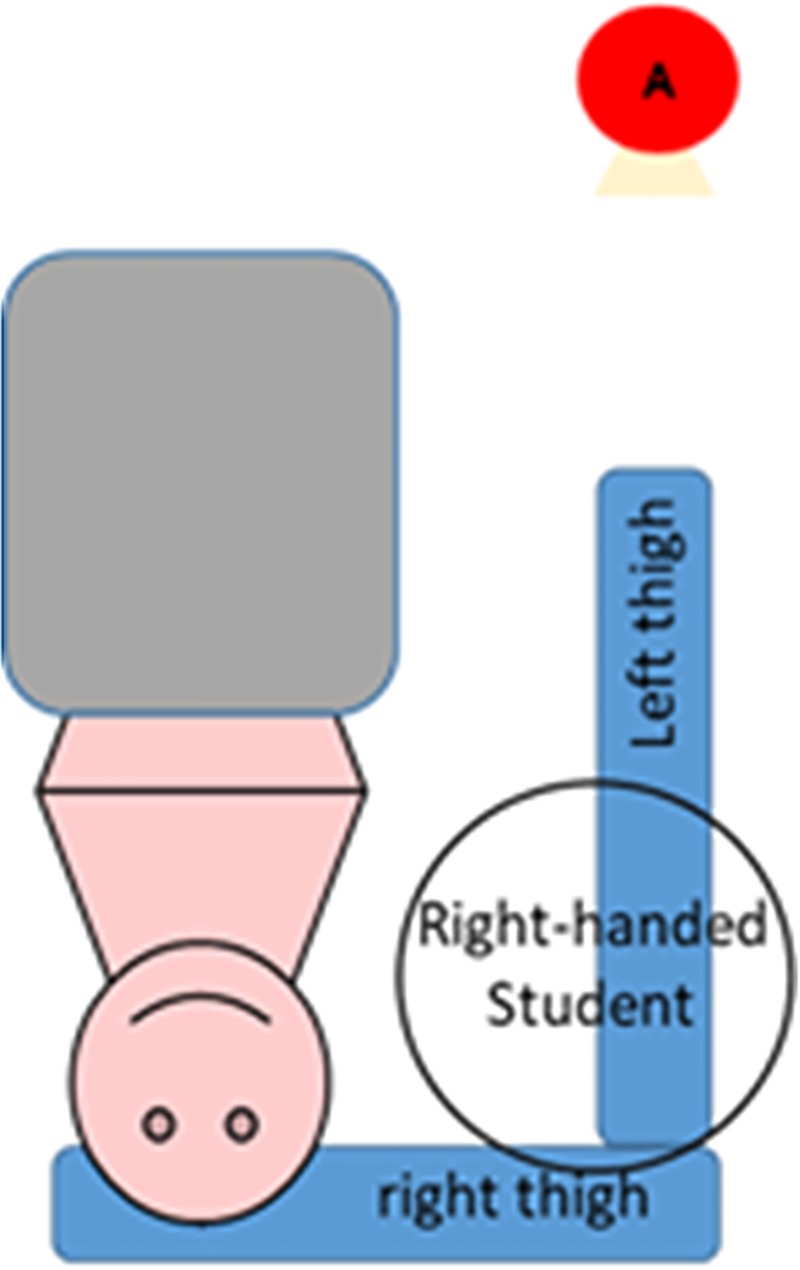
The point at which pictures of right-handed operators were taken (A).

**Fig 3:**
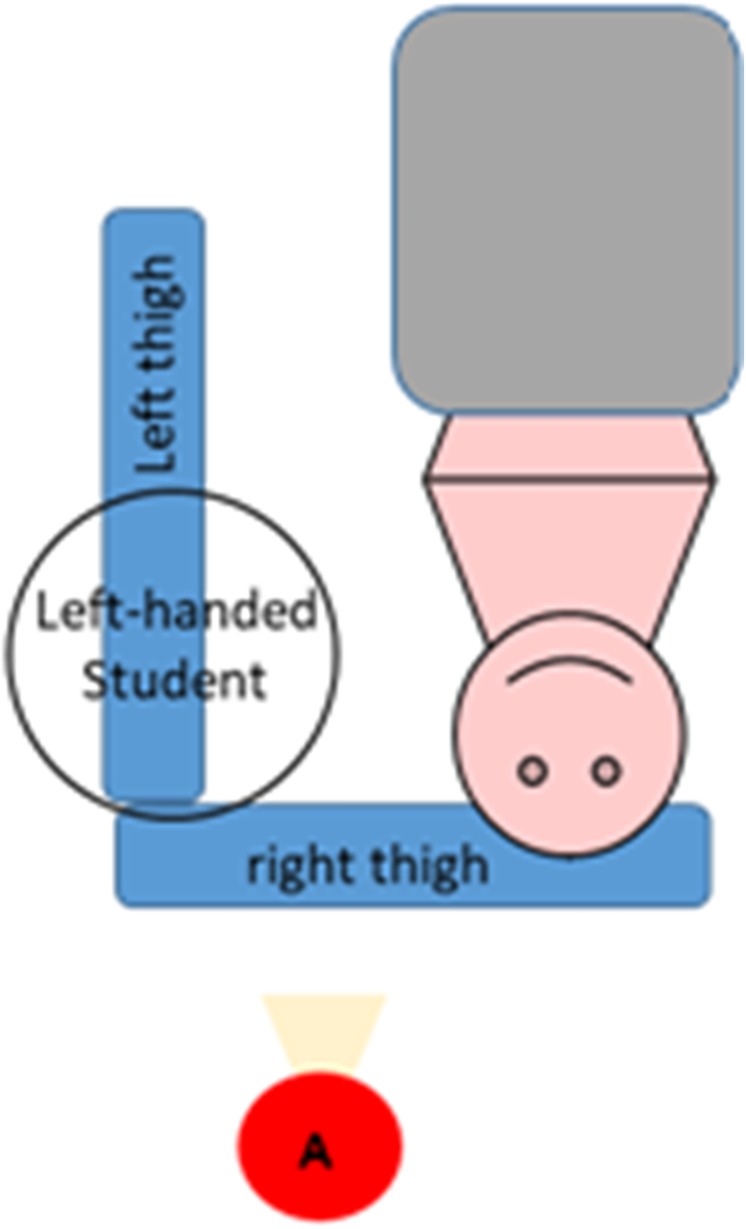
The point at which pictures of left-handed operators were taken (A).

To evaluate the angulation of the subjects’ necks in a neutral posture, photographs were taken during the visual acuity exam. A digital camera placed on a tripod was used for this purpose. The position of the camera and the tripod provided a lateral view of each subject. The points at which photographs were taken were established in the pilot study and are shown in Figs 2 and 3.

**Fig 4:**
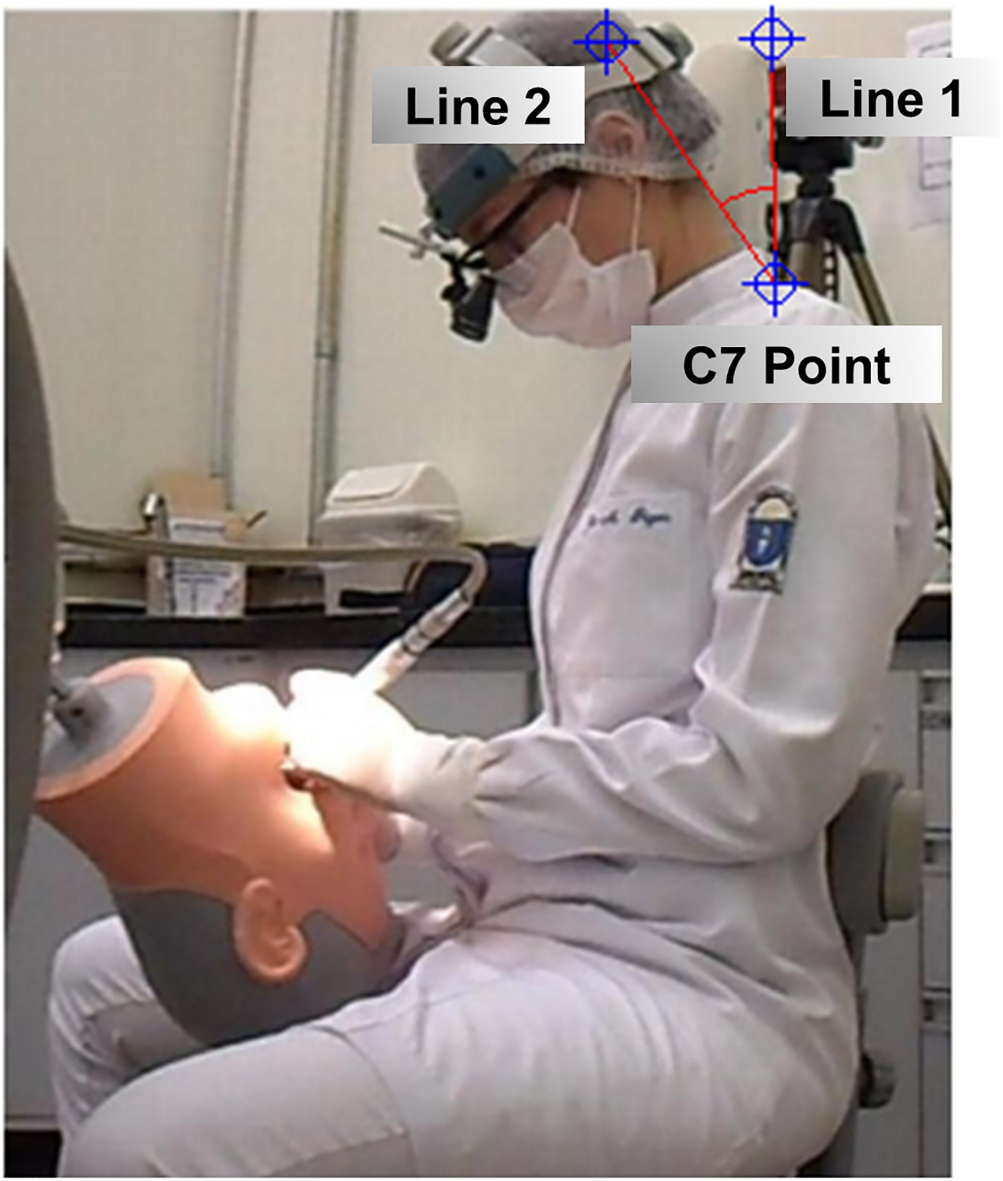
Neck angulation measurement.

Neck angulation was measured using a local posture evaluation software known as Software para Avaliação Postural, version 0.69 (Laboratory for Biomechanics and Motor Control Federal University of ABC [UFABC], São Bernardo do Campo, São Paulo State, Brazil). In this step, the seventh cervical vertebra was used as the reference (Point 1) for the two lines drawn. Line 1 was a vertical line representing the neck at a neutral position, while Line 2 was an oblique line from C7 that ran along the nape of the neck and followed the inclination of the neck (Fig 4). All of the measurements were obtained by a single trained researcher and duly calibrated (ρ = 0.88).

### Statistical analysis

The data from each graduating class evaluated of the dentistry program (second-, third-, fourth-, and fifth-year students) were analyzed independently and a descriptive statistical analysis was performed. Assumptions of normality were met (Sk = 0.55–0.80; Ku = 0.94–1.35); however, those of homoscedasticity were not (p<0.001). Because of this result, the two-factor analysis of variance (ANOVA) was combined with the Welch’s *t*-test and the Games-Howell post-hoc test for both the visual acuity analysis and the neck angulation analysis. All the statistical analyses were performed using the IBM SPSS Statistics software, version 20. The significance level adopted in this study was 5%.

## Results

Table 1 presents a summary of the ANOVA results, the mean, and the standard deviation of the visual acuity scores for students in the second through fifth years of the dentistry program organized by magnification device and distance.

**Table 1:** Summary of the ANOVA results, the mean, and the standard deviation of the visual acuity scores (C/°) of students in the second through fifth years of the dentistry program organized by the magnification system and distance adopted. Araraquara, 2017.

Year	Distance+	Magnification Device					Source of Variation++	SS	df	MS	F	*p*	η_p_^2^	π
		Naked Eye	Simple Loupe	Galilean Loupe	Keplerian Loupe	Microscope								
**2nd**	1	309.89±209.35Ab	32.81±106.94Bc	503.121±161.62	543.95 ±135.16Aa	477.601±183.84a	A	305864.86	1	305864.856	11.97	<0.01	0.03	0.93
	2	333.85±169.24Ab	285.37±114.16Ab	500.20±166.51	546.87±135.61Aa	-	B	8130856.72	4	2032714.18	79.56	<0.01	0.45	1.00
							A*B	981720.55	4	245430.14	9.60	<0.01	0.09	1.00
**3rd**	1	346.35±225.65Ab	22.10±70.38Bc	514.06±171.56Aa	535.93±133.69Aa	517.71±161.61a	A	177957.985	1	177957.985	7.42	<0.01	0.02	0.78
	2	343.75±153.73Ab	249.27±106.09Ab	515.00±156.32Aa	521.35±157.99Aa	-	B	9284445.91	4	2321111.47	96.83	<0.01	0.50	1.00
							A*B	858584.61	4	214646.15	8.95	<0.01	0.08	1.00
**4th**	1	251.56±223.95Ab	21.87±102.05Bc	510.41±143.95Aa	444.79±243.58Aa	528.64±153.80a	A	217591.93	1	217591.93	6.86	0.01	0.02	0.74
	2	273.96±161.05Ab	227.50±149.73Ab	522.70±132.20Aa	452.29±238.03Aa	-	B	9769578.60	4	2442394.65	76.98	<0.01	0.44	1.00
							A*B	161619.47	4	161619.47	5.09	<0.01	0.05	0.97
**5th**	1	229.69±200.57Ab	10.94±38.90Bc	503.12±161.61Aa	543.23±109.47Aa	583.33±0.00a	A	396805.09	1	396805.09	23.26	<0.01	0.06	1.00
	2	284.75±186.89Ab	273.96±136.12Ab	497.91±158.04Aa	545.31±111.03Aa	-	B	12087398.95	4	3021849.74	177.12	<0.01		1.00
							A*B	104807.52	4	262017.63	15.36	<0.01	0.13	1.00

+1 = standardized distance; 2 = comfortable distance;

++A = distances; B = magnification device;

the Games-Howell post-hoc test;;A.a: uppercase letters = rows; lowercase letters = columns

A significant difference in visual acuity was found between the “magnification device” and “distance” factors in each of the graduating classes analyzed. At a standardized distance, the Keplerian loupe, the Galilean loupe, and the operating microscope all provided greater visual acuity. At a subjectively comfortable distance, the Keplerian and Galilean loupes produced the best visual acuity. When simple loupes were used, visual acuity differed significantly between the standardized and comfortable distances.

Table 2 provides a summary of the ANOVA, the mean, and the standard deviation of the second- through fifth-year dentistry students’ neck angles during the tests with the miniature Snellen charts organized by magnification system and distance.

**Table 2:** Summary of the ANOVA, the mean, and the standard deviation of the second- through fifth-year dentistry students’ neck angles (°) during tests with miniature Snellen eye charts organized by magnification system and distance. Araraquara, 2017.

Year	Distance+	Magnification Device					Source of Variation++	SS	df	MS	F	*p*	η_p_^2^	π
		Naked Eye	Simple Loupe	Galilean Loupe	Keplerian Loupe	Microscope								
**2nd**	1	39.33±10.73Ba	37.31±8.95Ba	36.54±10.35Aa	32.83±7.77Aab	27.43±7.63b	A	6260.84	1	6260.84	56.72	<0.01	0.13	1.00
	2	56.08±17.72Aa	58.71±11.62Aa	36.53±10.35Ab	33.50±8.21Ab	-	B	26928.55	4	6732.14	60.99	<0.01	0.38	1.00
							A*B	9023.22	4	2255.80	20.44	<0.01	0.17	1.00
**3rd**	1	36.21±9.85Ba	36.98±11.95Ba	36.86±9.14Aa	33.94±9.74Aa	29.46±9.65a	A	5917.61	1	5917.61	54.41	<0.01	0.12	1.00
	2	54.60±12.64Aa	55.46±10.87Aa	38.20±10.41Ab	34.19±9.83Ab	-	B	16763.70	4	4190.92	38.53	<0.01	0.28	1.00
							A*B	7712.49	4	1928.12	17.73	<0.01	0.15	1.00
**4th**	1	35.72±8.02Ba	32.90±9.04Ba	38.29±9.63Aa	30.74±8.15Ab	27.25±9.12b	A	5882.89	1	5882.89	79.97	<0.01	0.17	1.00
	2	50.96±10.79Aa	54.58±8.44Aa	39.01±9.59Ab	31.44±7.98Ac	-	B	17455.18	4	4363.79	59.32	<0.01	0.38	1.00
							A*B	8186.07	4	2046.52	27.82	<0.01	0.22	1.00
**5th**	1	36.45±8.45Ba	35.61±9.27Ba	37.55±8.66Aa	35.90±6.77Aa	24.18±6.73b	A	7563.78	1	7563.78	91.60	<0.01	0.19	1.00
	2	56.51±13.55Aa	56.59±10.88Aa	39.72±10.33Ab	35.19±6.90Ab	-	B	27013.21	4	6753,30	81.78	<0.01	0.46	1.00
							A*B	9376.51	4	2344.13	28.39	<0.01	0.23	1.00

+1 = standardized distance; 2 = comfortable distance;

++A = distances; B = magnification device;

the Games-Howell post-hoc test;;A.a: uppercase letters = rows; lowercase letters = columns

A significant difference in neck angulation was found between the “magnification system” and “distance” factors in each of the graduating classes analyzed.

Among students in the second and fourth years of the program, the operating microscope and the Keplerian loupe used at a standardized distance resulted in less angulation of the neck. At a subjectively comfortable distance, the use of the Galilean and Keplerian loupes resulted in less angulation among the students in these graduating classes. Angulation of the neck was greater when the simple loupes were used at a subjectively comfortable distance, as well as when subjects participated in the experiment without the use of a magnification device (under the naked eye).

Among third-year students, the lowest angulation of the neck was measured when the Galilean and Keplerian loupes were used at a comfortable distance; at the standardized distance, the angulations did not differ significantly between the devices. Angulation of the neck was greater when the simple loupes were used at a subjectively comfortable distance, as well as when third-year students participated in the experiment without the use of a magnification device.

Among fifth-year students, the lowest angulation of the neck was measured with operating microscopes were used. At a subjectively comfortable distance, the Galilean and Keplerian loupes resulted in the lowest angulation. Angulation of the neck was greater when the simple loupes were used at a subjectively comfortable distance, as well as when subjects participated in the experiment without the use of a magnification device.

## Discussion

The recommendation of the use of magnification devices in educational settings assumes that these devices have been previously evaluated with regard to students’ visual acuity and working posture [10]. However, scientific research into this topic is scarce [1,10,13]. Thus, this study evaluated the effect of different magnification devices on dentistry students’ visual acuity and posture, with a particular emphasis on the angulation of the neck.

In the visual acuity tests, the use of the Galilean loupe, the Keplerian loupe, and the operating microscope resulted in the highest visual acuity values among the students; the results did not differ significantly between the devices at a standardized distance. Eichenberger et al [10] and Eichenberger et al [22], meanwhile, found that the visual acuity scores obtained with the use of Keplerian loupes were significantly higher than those obtained with Galilean loupes. This inconsistency between the studies may be a consequence of the devices’ different magnification powers or of the distances between the subjects’ eyes and the patients’ mouths established in each experiment. Eichenberger et al [10] and Eichenberger et al [22] used Galilean loupes at 2.5x magnification and Keplerian loupes at 4.3x magnification, thus producing a substantial difference in magnification power between the two that was not present in the current study. In addition, the operator-patient distance established by these authors depended on the magnification system used and ranged from 25 cm to 40 cm. In the current study, the operator-patient distance was set at approximately 35 cm for all of the systems tested.

As mentioned previously, the highest visual acuity scores were found when the Galilean and Keplerian lenses were used. Though the magnification power of the simple loupe was similar to that of the Galilean loupe (3.5x), the visual acuity that it provided was lower than that of the Galilean loupe among students from all of the graduating classes. Eichenberger et al [10] also found that, even at distances considered comfortable by the operators, the use of a simple loupe resulted in lower visual acuity scores than the other systems they evaluated. When the angulation of the neck was measured at each student’s subjectively comfortable distance, the degree of inclination was lowest overall when the Galilean and Keplerian loupes were used. The highest angles were found when the simple loupe was used and when no magnification device was used (under the naked eye).

At a standardized distance, second- and fourth-year students were found to have the lowest angulations of the neck when the Keplerian loupe and the operating microscope were used. Third-year students exhibited no differences in angulation between the devices, and fifth-year students had the lowest angulations when operating microscopes were used.

Students from all of the graduating classes therefore experienced low angulation of the neck with the use of the operating microscope. This result may have occurred because, once adjusted, this microscope prevents forward inclination of the neck and, as a consequence, any further angulation [22]. It is important to note that, among second- and fourth-year students, the angulation produced when the Keplerian loupe was used was similar to that produced with the use of the operating microscope. Due to the depth of the visual field, the Keplerian lens is less capable of focusing on objects. Operators therefore lose focus easily and with minimal movement of the head and are thus prevented from moving the neck once focus is obtained [18]. The use of Galilean and Keplerian loupes and of the operating microscope did not result in a deviation from the ideal neck angulation of 0 to 10° [25], however, these devices promote a significant decrease in angular deviation. These findings demonstrate that magnification may aid in decreasing dental surgeons’ risks of developing musculoskeletal disorders. Though the devices tested reduced the angular deviation of the neck, students are still at risk [25]. For this reason, additional studies involving these and other similar devices must be performed in order to determine ways to further decrease the angular deviation of the neck until risk to students is prevented completely.

In the current study, the visual acuity produced by the naked eye was found to be consistent whether at a comfortable or standardized distance. However, angulation of the neck differed significantly between these two distances, a finding which shows that, when operators were able to choose their working distance from the patient, they chose to work closer to the operating field and thus increase the angulation of the neck. This finding reinforces the importance of working at a distance of 30 cm to 40 cm from the patient’s mouth when no magnification systems are used. According to Saito et al [26], Porto [21], and Valachi [15], this distance provides an adequate angle for visibility of the objects in question and should therefore be maintained. At other angles, neck muscles must remain tense for operators to obtain adequate angles for visibility [26]. The students’ choice to move closer to the operating field may be due to the belief that proximity to a given object results in improved visibility of that object [27]. However, the data obtained in this study show that visual acuity did not improve with increased proximity to the operating field.

Though simple loupes were able to increase visual acuity at a subjectively comfortable distance, they made it difficult for the students to maintain adequate neck posture, and angulation increased significantly. It is also important to note that, of the magnification devices tested, the simple loupes produced the lowest visual acuity at the standardized distance. Thus, though simple lopes are low-cost and easily accessible options for magnifying the operating field, the focal distance they provide is limited, and they may have negative effects on operators’ occupational health [28].

The use of Keplerian and Galilean loupes resulted in higher visual acuity scores among students in all of the graduating classes, and these scores did not differ significantly between the standardized and comfortable distances. These results are consistent with those of prior studies [10,16,22]. The current study also found that angulation of the neck did not differ significantly between the standardized and comfortable distances when these two lenses were used. This finding suggests that, when these lenses are used, a comfortable operator-patient distance can be maintained. Branson et al [29] also found beneficial effects for dental surgeons’ head angulation with the use of magnifying lenses.

It is important to note that all of the magnification devices evaluated herein (and the Keplerian and Galilean loupes in particular) provided improved visual acuity for all of the study subjects without increasing neck angulation, regardless of the student’s year of enrollment in the dentistry program. Thus, these devices may be implemented as early as the pre-clinical phase of dentistry degree programs so that students may develop their professional motor skills with a magnified field.

A limitation of this study was the inability to analyze and subsequently compare visual acuity provided by the microscope at a subjective comfortable distance as chosen by each study subject. The operating microscope could not be evaluated at a variety of distances due to its physical characteristics. Additionally, due to the differences in loupe configuration, the magnification powers could not be the same. Future research should evaluate students’ posture while using magnification devices during laboratory and clinical procedures.

## Conclusion

At both a standardized distance and a comfortable distance, the Galilean and Keplerian magnification systems provided the best visual acuity and the lowest angulation of the operator’s neck. At a standardized distance of 30 cm to 40 cm, the operating microscopes produced similar results.

## Supporting information

S1 Table Data regarding to visual acuity and neck angulation, according to year of the course. (PDF)
