# Supplementary material for: Effect of magnification devices on dental students’ visual acuity
Source: PLoS One. 2019 Mar 27;14(3):e0212793. doi: 10.1371/journal.pone.0212793 (PMC6436677; doi:10.1371/journal.pone.0212793)
Supplement: S1 Table — (PDF) [file pone.0212793.s001.pdf]

ID	Loupe	Distance	2 year Visual Acuity	Neck angulation
1	1	1	291,66	34,8
2	1	1	583,33	31
3	1	1	583,33	32,4
4	1	1	583,33	17,5
5	1	1	583,33	31,7
6	1	1	583,33	33,7
7	1	1	291,66	40,6
8	1	1	0,00	68,9
9	1	1	583,33	42,3
10	1	1	291,66	57,8
11	1	1	291,66	42,6
12	1	1	291,66	31,5
13	1	1	0,00	35
14	1	1	583,33	42,5
15	1	1	291,66	33,6
16	1	1	291,66	38,6
17	1	1	145,83	37,6
18	1	1	145,83	38,6
19	1	1	145,83	33,3
20	1	1	291,66	51,4
21	1	1	0,00	45,8
22	1	1	291,66	56,2
23	1	1	0,00	43,7
24	1	1	291,66	30,2
25	1	1	0,00	23,2
26	1	1	0,00	28,7
27	1	1	583,33	61,3
28	1	1	291,66	37,7
29	1	1	291,66	56,9
30	1	1	145,83	47,9
31	1	1	145,83	39,2
32	1	1	291,66	31,3
33	1	1	583,33	27
34	1	1	0,00	37,5
35	1	1	583,33	42,8
36	1	1	291,66	32,5
37	1	1	291,66	46
38	1	1	583,33	48,8
39	1	1	291,66	29,3
40	1	1	583,33	31,7
1	1	2	100,00	49,6
2	1	2	0,00	28,2
3	1	2	583,33	54,8
4	1	2	416,67	50,3

5	1	2	583,33	31,7
6	1	2	516,67	33,7
7	1	2	233,33	40,6
8	1	2	0,00	68,9
9	1	2	316,67	72,4
10	1	2	350,00	68,9
11	1	2	316,67	63,9
12	1	2	316,67	72,7
13	1	2	500,00	47,1
14	1	2	583,33	42,5
15	1	2	350,00	37,3
16	1	2	366,67	43,4
17	1	2	300,00	37,6
18	1	2	0,00	62,9
19	1	2	350,00	46,7
20	1	2	400,00	96,3
21	1	2	100,00	64,4
22	1	2	400,00	77,8
23	1	2	316,67	87,8
24	1	2	400,00	69,8
25	1	2	516,67	50,6
26	1	2	0,00	66,5
27	1	2	433,33	62,8
28	1	2	250,00	76,9
29	1	2	383,33	82,2
30	1	2	383,33	79,3
31	1	2	383,33	65,4
32	1	2	416,67	50,8
33	1	2	583,33	27
34	1	2	95,83	40,2
35	1	2	316,67	73,2
36	1	2	158,33	33,6
37	1	2	383,33	67,4
38	1	2	583,33	48,8
39	1	2	350,00	34,7
40	1	2	316,67	63,5
1	2	1	0,00	31,3
2	2	1	0,00	20,9
3	2	1	0,00	41,4
4	2	1	0,00	26,6
5	2	1	583,33	36,6
6	2	1	0,00	34,8
7	2	1	0,00	42,3
8	2	1	0,00	51,8
9	2	1	0,00	39,6
10	2	1	0,00	40,9

11	2	1	0,00	35,3
12	2	1	0,00	40,3
13	2	1	0,00	31,4
14	2	1	0,00	38,5
15	2	1	0,00	36,1
16	2	1	0,00	26,2
17	2	1	0,00	31,5
18	2	1	0,00	38,3
19	2	1	145,83	40,1
20	2	1	145,83	57,2
21	2	1	0,00	43,8
22	2	1	0,00	55,7
23	2	1	0,00	46,8
24	2	1	0,00	33,9
25	2	1	0,00	18
26	2	1	0,00	30,1
27	2	1	0,00	58,7
28	2	1	291,66	37,6
29	2	1	0,00	35,1
30	2	1	0,00	36,9
31	2	1	0,00	29,7
32	2	1	0,00	38,9
33	2	1	0,00	36,4
34	2	1	145,83	34,6
35	2	1	0,00	48,3
36	2	1	0,00	27,8
37	2	1	0,00	43,4
38	2	1	0,00	33,6
39	2	1	0,00	26,6
40	2	1	0,00	35,4
1	2	2	233,33	60,2
2	2	2	333,33	50,8
3	2	2	400,00	51
4	2	2	183,33	61,1
5	2	2	500,00	41,1
6	2	2	450,00	49,6
7	2	2	158,33	71,9
8	2	2	316,67	71,4
9	2	2	350,00	68,4
10	2	2	350,00	79,7
11	2	2	300,00	59,9
12	2	2	283,33	68,6
13	2	2	416,67	42
14	2	2	200,00	75,2
15	2	2	350,00	50,2
16	2	2	333,33	51,2

17	2	2	300,00	59,2
18	2	2	0,00	62,9
19	2	2	350,00	48,1
20	2	2	400,00	69,1
21	2	2	400,00	62,9
22	2	2	350,00	77,6
23	2	2	250,00	80,9
24	2	2	183,33	62,6
25	2	2	283,33	51,1
26	2	2	300,00	60,1
27	2	2	350,00	60,9
28	2	2	250,00	65,4
29	2	2	283,33	65,2
30	2	2	366,67	55,4
31	2	2	350,00	45,9
32	2	2	316,67	55,2
33	2	2	316,67	37,4
34	2	2	158,33	34,6
35	2	2	316,67	62,3
36	2	2	141,67	43,9
37	2	2	141,67	69,5
38	2	2	416,67	61,1
39	2	2	0,00	43,3
40	2	2	31,67	61,8
1	3	1	583,33	27,8
2	3	1	583,33	20,6
3	3	1	583,33	40
4	3	1	583,33	1,1
5	3	1	583,33	48,4
6	3	1	583,33	42,8
7	3	1	583,33	36,3
8	3	1	291,66	35,9
9	3	1	583,33	35,4
10	3	1	583,33	56,1
11	3	1	583,33	33,8
12	3	1	583,33	39,4
13	3	1	583,33	33,8
14	3	1	583,33	42,2
15	3	1	0,00	34,7
16	3	1	583,33	32,6
17	3	1	583,33	30,1
18	3	1	583,33	36,4
19	3	1	583,33	38,2
20	3	1	291,66	45
21	3	1	583,33	41,8
22	3	1	291,66	66,2

23	3	1	583,33	37,2
24	3	1	291,66	37,7
25	3	1	583,33	35,1
26	3	1	291,66	44,5
27	3	1	583,33	49,2
28	3	1	291,66	35,6
29	3	1	291,66	45,3
30	3	1	583,33	34
31	3	1	583,33	29,7
32	3	1	583,33	31,3
33	3	1	583,33	35,3
34	3	1	0,00	17,3
35	3	1	583,33	26,3
36	3	1	583,33	32,6
37	3	1	583,33	34,8
38	3	1	583,33	42,9
39	3	1	583,33	36
40	3	1	583,33	38,3
1	3	2	583,33	27,8
2	3	2	583,33	20,6
3	3	2	583,33	40
4	3	2	583,33	1,1
5	3	2	583,33	48,4
6	3	2	583,33	42,8
7	3	2	583,33	36,3
8	3	2	291,66	35,9
9	3	2	583,33	35,4
10	3	2	583,33	56,1
11	3	2	583,33	33,8
12	3	2	583,33	39,4
13	3	2	583,33	33,8
14	3	2	583,33	42,2
15	3	2	0,00	34,7
16	3	2	583,33	32,6
17	3	2	583,33	30,1
18	3	2	583,33	36,4
19	3	2	583,33	38,2
20	3	2	291,66	45
21	3	2	583,33	41,8
22	3	2	291,66	66,2
23	3	2	583,33	37,21
24	3	2	291,66	37,7
25	3	2	583,33	35,1
26	3	2	291,66	44,5
27	3	2	583,33	49,2
28	3	2	175,00	35,6

29	3	2	291,66	45,3
30	3	2	583,33	34
31	3	2	583,33	29,7
32	3	2	583,33	31,3
33	3	2	583,33	35,3
34	3	2	0,00	17,3
35	3	2	583,33	26,3
36	3	2	583,33	32,6
37	3	2	583,33	34,8
38	3	2	583,33	42,9
39	3	2	583,33	36
40	3	2	583,33	38,3
1	4	1	583,33	24,9
2	4	1	583,33	34,6
3	4	1	583,33	34,5
4	4	1	583,33	33,5
5	4	1	583,33	16,2
6	4	1	583,33	32,2
7	4	1	583,33	23,5
8	4	1	583,33	32,3
9	4	1	583,33	36,7
10	4	1	583,33	43
11	4	1	583,33	37,7
12	4	1	583,33	33,4
13	4	1	583,33	35,4
14	4	1	583,33	38,5
15	4	1	583,33	30,9
16	4	1	583,33	29,8
17	4	1	583,33	26,6
18	4	1	583,33	32,8
19	4	1	466,67	30,5
20	4	1	583,33	47,2
21	4	1	583,33	31,2
22	4	1	583,33	53,4
23	4	1	583,33	38,1
24	4	1	583,33	27,5
25	4	1	583,33	30,2
26	4	1	583,33	24,3
27	4	1	583,33	42,4
28	4	1	583,33	27,1
29	4	1	583,33	45
30	4	1	583,33	22,8
31	4	1	583,33	26,8
32	4	1	583,33	31,1
33	4	1	583,33	29,8
34	4	1	291,66	15,4

35	4	1	583,33	32,1
36	4	1	0,00	41,3
37	4	1	0,00	36,4
38	4	1	583,33	37,4
39	4	1	583,33	39,7
40	4	1	583,33	27
1	4	2	583,33	24,9
2	4	2	583,33	34,6
3	4	2	583,33	34,5
4	4	2	583,33	33,5
5	4	2	583,33	16,2
6	4	2	583,33	32,2
7	4	2	583,33	23,5
8	4	2	583,33	32,3
9	4	2	583,33	36,7
10	4	2	583,33	43
11	4	2	583,33	37,7
12	4	2	583,33	33,4
13	4	2	583,33	35,4
14	4	2	583,33	38,5
15	4	2	583,33	30,9
16	4	2	583,33	29,8
17	4	2	583,33	26,6
18	4	2	583,33	45,2
19	4	2	583,33	45,2
20	4	2	583,33	47,2
21	4	2	583,33	31,2
22	4	2	583,33	53,4
23	4	2	583,33	38,1
24	4	2	583,33	27,5
25	4	2	583,33	30,2
26	4	2	583,33	24,3
27	4	2	583,33	42,4
28	4	2	583,33	27,1
29	4	2	583,33	45
30	4	2	583,33	22,8
31	4	2	583,33	26,8
32	4	2	583,33	31,1
33	4	2	583,33	29,8
34	4	2	291,66	15,4
35	4	2	583,33	32,1
36	4	2	0,00	41,3
37	4	2	0,00	36,4
38	4	2	583,33	37,4
39	4	2	583,33	39,7
40	4	2	583,33	27

1	5	1	583,33	21,5
2	5	1	583,33	23,9
3	5	1	583,33	25,9
4	5	1	583,33	21,4
5	5	1	583,33	36,1
6	5	1	583,33	37,8
7	5	1	583,33	21,2
8	5	1	583,33	20,2
9	5	1	583,33	26,5
10	5	1	583,33	21,3
11	5	1	583,33	30,6
12	5	1	583,33	30,5
13	5	1	583,33	17,3
14	5	1	583,33	29,5
15	5	1	583,33	26,6
16	5	1	583,33	19,9
17	5	1	583,33	22,6
18	5	1	583,33	20,1
19	5	1	583,33	15,6
20	5	1	583,33	26,6
21	5	1	583,33	25,5
22	5	1	145,83	30
23	5	1	0,00	23,5
24	5	1	291,66	21,2
25	5	1	583,33	23,8
26	5	1	291,66	31,8
27	5	1	583,33	34,2
28	5	1	583,33	14,8
29	5	1	583,33	30
30	5	1	583,33	26,4
31	5	1	583,33	24,7
32	5	1	583,33	26,4
33	5	1	291,66	28,8
34	5	1	583,33	33,6
35	5	1	0,00	49,3
36	5	1	291,66	32,8
37	5	1	291,66	39,3
38	5	1	145,83	47,2
39	5	1	291,66	23,6
40	5	1	145,83	35,4

3 year

ID	Loupe	Distance	Visual Acuity	Neck angulation
1	1	1	291,66	27,9
2	1	1	583,33	28,5
3	1	1	291,66	23

4	1	1	583,33	39
5	1	1	583,33	44,4
6	1	1	291,66	32,5
7	1	1	0	36,6
8	1	1	583,33	24,7
9	1	1	291,66	17,9
10	1	1	291,66	24
11	1	1	0	33,9
12	1	1	145,83	37
13	1	1	145,83	25,6
14	1	1	291,66	21,7
15	1	1	583,33	45,3
16	1	1	291,66	38,5
17	1	1	583,33	34,9
18	1	1	583,33	31,7
19	1	1	583,33	50,2
20	1	1	145,83	31,4
21	1	1	583,33	46
22	1	1	583,33	57,9
23	1	1	583,33	44,5
24	1	1	0	42,4
25	1	1	0	29,2
26	1	1	0	55,8
27	1	1	145,83	36,9
28	1	1	583,33	37,9
29	1	1	583,33	50,5
30	1	1	291,66	25,9
31	1	1	145,83	30,7
32	1	1	0	33,7
33	1	1	145,83	34,3
34	1	1	291,66	36,2
35	1	1	583,33	44,1
36	1	1	291,66	30,5
37	1	1	145,83	32,7
38	1	1	583,33	31,5
39	1	1	583,33	40,1
40	1	1	583,33	58,9
1	1	2	550	53,2
2	1	2	200	34
3	1	2	200	50,5
4	1	2	416,6666667	60
5	1	2	383,3333333	68,3
6	1	2	500	63,8
7	1	2	0	65,2
8	1	2	416,6666667	26,6
9	1	2	200	58,2

10	1	2	300	61,6
11	1	2	0	52,7
12	1	2	466,6666667	60,9
13	1	2	233,3333333	52,8
14	1	2	333,3333333	53
15	1	2	283,3333333	79,4
16	1	2	333,3333333	64
17	1	2	583,33	34,9
18	1	2	333,3333333	33,7
19	1	2	400	60,3
20	1	2	333,3333333	61
21	1	2	125	69
22	1	2	400	68
23	1	2	433,3333333	69,1
24	1	2	125	70,22
25	1	2	500	32,1
26	1	2	500	55,8
27	1	2	500	46,8
28	1	2	583,33	37,9
29	1	2	583,33	50,5
30	1	2	333,3333333	53,1
31	1	2	158,3333333	30,7
32	1	2	366,6666667	48,3
33	1	2	150	66,9
34	1	2	366,6666667	54,6
35	1	2	400	46,7
36	1	2	500	50,4
37	1	2	125	66,5
38	1	2	333,3333333	48,2
39	1	2	416,6666667	60
40	1	2	383,3333333	65,2
1	2	1	0	31,4
2	2	1	291,66	22,3
3	2	1	145,83	30,2
4	2	1	0	30
5	2	1	291,66	51,8
6	2	1	0	56,3
7	2	1	0	23,7
8	2	1	0	55,3
9	2	1	0	42,6
10	2	1	0	32,1
11	2	1	0	16,8
12	2	1	0	35,5
13	2	1	0	30,5
14	2	1	0	12
15	2	1	0	42

16	2	1	0	29,5
17	2	1	0	36,2
18	2	1	0	24,3
19	2	1	0	26,6
20	2	1	0	23,7
21	2	1	0	38
22	2	1	0	36,9
23	2	1	0	41,7
24	2	1	0	57,3
25	2	1	0	17,9
26	2	1	0	65,9
27	2	1	145,83	54,8
28	2	1	0	43,8
29	2	1	0	42,6
30	2	1	0	35,9
31	2	1	0	28,7
32	2	1	0	49,2
33	2	1	0	34,7
34	2	1	0	36,5
35	2	1	0	38,2
36	2	1	0	36
37	2	1	0	50
38	2	1	0	35,8
39	2	1	0	41,5
40	2	1	9	41,1
1	2	2	0	51,6
2	2	2	400	35,3
3	2	2	100	35,1
4	2	2	333,3333333	44,2
5	2	2	200	66,1
6	2	2	383,3333333	74,1
7	2	2	95,83333333	56,1
8	2	2	300	53,9
9	2	2	200	60,7
10	2	2	191,6666667	59,4
11	2	2	383,3333333	59,5
12	2	2	316,6666667	67,4
13	2	2	316,6666667	55,8
14	2	2	350	39,6
15	2	2	158,3333333	66
16	2	2	300	69,7
17	2	2	266,6666667	58,9
18	2	2	283,3333333	52,4
19	2	2	200	53,1
20	2	2	300	53,3
21	2	2	283,3333333	57,2

22	2	2	183,3333333	55,7
23	2	2	350	55,6
24	2	2	350	29,4
25	2	2	283,3333333	39,1
26	2	2	300	65,9
27	2	2	0	68,7
28	2	2	283,3333333	68
29	2	2	283,3333333	69,2
30	2	2	366,6666667	38,8
31	2	2	158,3333333	39,1
32	2	2	366,6666667	54,8
33	2	2	150	60,8
34	2	2	150	55
35	2	2	283,3333333	51,3
36	2	2	200	55,2
37	2	2	350	58,3
38	2	2	100	57,2
39	2	2	350	69,6
40	2	2	100	57,2
1	3	1	291,66	46,5
2	3	1	145,83	34,9
3	3	1	145,83	31,3
4	3	1	145,83	31,7
5	3	1	583,33	40,1
6	3	1	583,33	56
7	3	1	0	36
8	3	1	583,33	19,8
9	3	1	583,33	23
10	3	1	583,33	30,3
11	3	1	583,33	27,6
12	3	1	583,33	35,8
13	3	1	583,33	35,9
14	3	1	583,33	22,3
15	3	1	583,33	36,2
16	3	1	583,33	39,3
17	3	1	583,33	42,2
18	3	1	583,33	23,7
19	3	1	583,33	32,5
20	3	1	583,33	25,1
21	3	1	583,33	44,5
22	3	1	583,33	38,5
23	3	1	583,33	41,5
24	3	1	583,33	27,7
25	3	1	583,33	21,2
26	3	1	583,33	51,2
27	3	1	0	51,3

28	3	1	583,33	49,8
29	3	1	583,33	46,7
30	3	1	583,33	40,9
31	3	1	583,33	36,3
32	3	1	583,33	38,7
33	3	1	583,33	41,3
34	3	1	583,33	38,1
35	3	1	583,33	30
36	3	1	583,33	42,5
37	3	1	583,33	51,4
38	3	1	583,33	41
39	3	1	583,33	28,8
40	3	1	583,33	42,6
1	3	2	500	65,1
2	3	2	116,6666667	49,6
3	3	2	145,83	44,9
4	3	2	145,83	31,7
5	3	2	583,33	45,6
6	3	2	583,33	56
7	3	2	0	36
8	3	2	583,33	19,8
9	3	2	583,33	23
10	3	2	583,33	30,3
11	3	2	583,33	27,6
12	3	2	583,33	35,8
13	3	2	583,33	35,9
14	3	2	208,3333333	22,3
15	3	2	516,6666667	41,8
16	3	2	583,33	39,3
17	3	2	583,33	42,2
18	3	2	383,3333333	23,7
19	3	2	583,33	28,4
20	3	2	583,33	25,1
21	3	2	583,33	44,5
22	3	2	583,33	38,5
23	3	2	583,33	41,5
24	3	2	583,33	27,7
25	3	2	583,33	21,2
26	3	2	583,33	51,2
27	3	2	500	51,3
28	3	2	583,33	49,8
29	3	2	583,33	46,7
30	3	2	583,33	40,9
31	3	2	583,33	36,3
32	3	2	583,33	38,7
33	3	2	583,33	41,3

34	3	2	583,33	38,1
35	3	2	583,33	30
36	3	2	583,33	42,5
37	3	2	583,33	51,4
38	3	2	583,33	41
39	3	2	583,33	28,8
40	3	2	583,33	42,6
1	4	1	583,33	29,8
2	4	1	145,83	34,2
3	4	1	583,33	39,9
4	4	1	583,33	35,1
5	4	1	583,33	33,2
6	4	1	583,33	44,2
7	4	1	583,33	38,4
8	4	1	583,33	20,2
9	4	1	583,33	19,9
10	4	1	0	35
11	4	1	583,33	30,7
12	4	1	291,66	50,4
13	4	1	583,33	32,5
14	4	1	583,33	25,7
15	4	1	583,33	24,1
16	4	1	583,33	24
17	4	1	583,33	43,4
18	4	1	583,33	19,9
19	4	1	583,33	25,7
20	4	1	583,33	29,6
21	4	1	583,33	30,7
22	4	1	583,33	48
23	4	1	583,33	29,7
24	4	1	583,33	27,1
25	4	1	583,33	21,7
26	4	1	291,66	39,3
27	4	1	583,33	45,7
28	4	1	583,33	57,1
29	4	1	583,33	40,8
30	4	1	291,66	41,9
31	4	1	583,33	41,7
32	4	1	583,33	38,9
33	4	1	583,33	44,1
34	4	1	583,33	35,1
35	4	1	583,33	21
36	4	1	583,33	18,5
37	4	1	583,33	25
38	4	1	583,33	25,8
39	4	1	583,33	45,6

40	4	1	583,33	44
1	4	2	583,33	29,8
2	4	2	145,83	34,2
3	4	2	583,33	39,9
4	4	2	583,33	35,1
5	4	2	583,33	33,2
6	4	2	0	44,2
7	4	2	583,33	38,4
8	4	2	583,33	20,2
9	4	2	583,33	19,9
10	4	2	0	35
11	4	2	583,33	30,7
12	4	2	291,66	50,4
13	4	2	583,33	42,4
14	4	2	583,33	25,7
15	4	2	583,33	24,1
16	4	2	583,33	24
17	4	2	583,33	43,4
18	4	2	583,33	19,9
19	4	2	583,33	25,7
20	4	2	583,33	29,6
21	4	2	583,33	30,7
22	4	2	583,33	48
23	4	2	583,33	29,7
24	4	2	583,33	27,1
25	4	2	583,33	21,7
26	4	2	291,66	39,3
27	4	2	583,33	45,7
28	4	2	583,33	57,1
29	4	2	583,33	40,8
30	4	2	291,66	41,9
31	4	2	583,33	41,7
32	4	2	583,33	38,9
33	4	2	583,33	44,1
34	4	2	583,33	35,1
35	4	2	583,33	21
36	4	2	583,33	18,5
37	4	2	583,33	25
38	4	2	583,33	25,8
39	4	2	583,33	45,6
40	4	2	583,33	44
1	5	1	583,33	25,4
2	5	1	145,83	23,3
3	5	1	145,83	21
4	5	1	583,33	38,3
5	5	1	145,83	19,8

6	5	1	583,33	33,1
7	5	1	583,33	28,4
8	5	1	583,33	14
9	5	1	583,33	36,7
10	5	1	583,33	27,8
11	5	1	583,33	41,2
12	5	1	583,33	44,5
13	5	1	583,33	41,4
14	5	1	583,33	24,9
15	5	1	583,33	7,6
16	5	1	583,33	25,3
17	5	1	583,33	40,2
18	5	1	583,33	16,5
19	5	1	583,33	32,4
20	5	1	583,33	16,9
21	5	1	583,33	29,8
22	5	1	583,33	39,8
23	5	1	583,33	25
24	5	1	583,33	34,7
25	5	1	145,83	17,7
26	5	1	583,33	47,2
27	5	1	0	40,4
28	5	1	291,66	42,9
29	5	1	583,33	35,2
30	5	1	583,33	36,6
31	5	1	583,33	36,6
32	5	1	583,33	21,7
33	5	1	583,33	31,6
34	5	1	583,33	30,5
35	5	1	583,33	26
36	5	1	583,33	24,7
37	5	1	583,33	22,1
38	5	1	583,33	16,5
39	5	1	583,33	20,8
40	5	1	583,33	40,1

			4 year	
**ID**	**Loupe**	**Distance**	**Visual Acuity**	**Neck angulation**
1	1	1	583,33	31,3
2	1	1	145,83	18,4
3	1	1	0,00	19,9
4	1	1	583,33	46,6
5	1	1	291,66	55,8
6	1	1	291,66	30,3
7	1	1	583,33	39,9
8	1	1	291,66	29,4
9	1	1	583,33	33,3
10	1	1	0,00	32,8
11	1	1	583,33	24,2
12	1	1	145,83	40,6
13	1	1	0,00	35,4
14	1	1	145,83	41,7
15	1	1	145,83	22,7
16	1	1	583,33	38,8
17	1	1	291,66	36,3
18	1	1	583,33	33,7
19	1	1	583,33	35,2
20	1	1	291,66	39
21	1	1	0,00	37,7
22	1	1	0,00	33,1
23	1	1	291,66	34
24	1	1	291,66	31,1
25	1	1	0,00	44,6
26	1	1	145,83	45,3
27	1	1	0,00	31,4
28	1	1	0,00	31,8
29	1	1	291,66	37,3
30	1	1	291,66	28,2
31	1	1	0,00	55,9
32	1	1	291,66	37,1
33	1	1	583,33	43,9
34	1	1	0,00	30,8
35	1	1	291,66	27,8
36	1	1	145,83	42,1
37	1	1	145,83	39,5
38	1	1	0,00	35,9
39	1	1	583,33	39,1
40	1	1	0,00	36,9
1	1	2	550,00	59,3
2	1	2	450,00	31,2
3	1	2	0,00	29,2
4	1	2	416,67	46,6

5	1	2	191,67	64,9
6	1	2	466,67	65,9
7	1	2	500,00	49
8	1	2	400,00	52,2
9	1	2	450,00	58,4
10	1	2	350,00	32,8
11	1	2	0,00	48,1
12	1	2	0,00	52,7
13	1	2	175,00	53,3
14	1	2	208,33	51
15	1	2	350,00	41,7
16	1	2	416,67	58,9
17	1	2	250,00	40,5
18	1	2	350,00	60,7
19	1	2	433,33	49,5
20	1	2	208,33	44,6
21	1	2	283,33	57,7
22	1	2	175,00	57,5
23	1	2	250,00	40,6
24	1	2	466,67	45,8
25	1	2	125,00	48,1
26	1	2	350,00	66,8
27	1	2	0,00	55,9
28	1	2	0,00	64,6
29	1	2	183,33	53,1
30	1	2	400,00	41,7
31	1	2	0,00	55,9
32	1	2	500,00	69,1
33	1	2	150,00	43,9
34	1	2	350,00	34,3
35	1	2	366,67	32,7
36	1	2	350,00	61,1
37	1	2	191,67	66,4
38	1	2	175,00	59,9
39	1	2	283,33	39,1
40	1	2	191,67	53,9
1	2	1	0,00	37,5
2	2	1	0,00	11,4
3	2	1	0,00	29
4	2	1	583,33	16,6
5	2	1	0,00	37,2
6	2	1	0,00	26,8
7	2	1	0,00	31,8
8	2	1	0,00	37,6
9	2	1	0,00	34,7
10	2	1	0,00	36,8

11	2	1	0,00	10,8
12	2	1	0,00	34
13	2	1	0,00	34
14	2	1	0,00	26,6
15	2	1	0,00	29,6
16	2	1	0,00	36,9
17	2	1	291,66	37,1
18	2	1	0,00	42,9
19	2	1	0,00	33,8
20	2	1	0,00	51,1
21	2	1	0,00	40,2
22	2	1	0,00	31,1
23	2	1	0,00	27
24	2	1	0,00	27,8
25	2	1	0,00	27,9
26	2	1	0,00	55,6
27	2	1	0,00	30,1
28	2	1	0,00	29,1
29	2	1	0,00	31,2
30	2	1	0,00	33,5
31	2	1	0,00	35,4
32	2	1	0,00	39,6
33	2	1	0,00	44,5
34	2	1	0,00	37,9
35	2	1	0,00	23
36	2	1	0,00	45
37	2	1	0,00	35,5
38	2	1	0,00	36,4
39	2	1	0,00	27,9
40	2	1	0,00	21
1	2	2	550,00	64,4
2	2	2	125,00	56,2
3	2	2	0,00	59,9
4	2	2	266,67	56,7
5	2	2	0,00	46,3
6	2	2	416,67	58
7	2	2	416,67	55,8
8	2	2	416,67	51,6
9	2	2	158,33	61,8
10	2	2	350,00	43
11	2	2	300,00	48,7
12	2	2	0,00	47,7
13	2	2	208,33	48,9
14	2	2	350,00	47,3
15	2	2	125,00	54,5
16	2	2	266,67	65,4

17	2	2	416,67	43,4
18	2	2	166,67	54
19	2	2	175,00	48,4
20	2	2	175,00	56,3
21	2	2	175,00	45,4
22	2	2	400,00	52
23	2	2	0,00	58,4
24	2	2	316,67	59,2
25	2	2	283,33	45,6
26	2	2	316,67	64,5
27	2	2	0,00	43
28	2	2	0,00	54,1
29	2	2	141,67	49,8
30	2	2	283,33	62,2
31	2	2	0,00	60,6
32	2	2	0,00	71
33	2	2	141,67	61,9
34	2	2	333,33	58,9
35	2	2	350,00	49,6
36	2	2	350,00	59
37	2	2	175,00	72,4
38	2	2	316,67	35,6
39	2	2	283,33	68
40	2	2	350,00	43,6
1	3	1	0,00	21
2	3	1	583,33	34,3
3	3	1	583,33	24
4	3	1	583,33	43,7
5	3	1	583,33	19,6
6	3	1	583,33	31
7	3	1	291,66	46,4
8	3	1	583,33	38,1
9	3	1	583,33	32,9
10	3	1	583,33	43,6
11	3	1	583,33	26,6
12	3	1	583,33	36
13	3	1	583,33	28,4
14	3	1	583,33	52,7
15	3	1	583,33	35,9
16	3	1	583,33	57,6
17	3	1	291,66	23,3
18	3	1	291,66	42,3
19	3	1	583,33	35,7
20	3	1	291,66	37,1
21	3	1	583,33	26,6
22	3	1	583,33	45,3

23	3	1	583,33	30,4
24	3	1	583,33	53
25	3	1	583,33	34,4
26	3	1	583,33	53,2
27	3	1	583,33	40,6
28	3	1	583,33	41,1
29	3	1	583,33	39
30	3	1	583,33	39,4
31	3	1	291,66	35,3
32	3	1	583,33	54,9
33	3	1	291,66	36
34	3	1	583,33	33,3
35	3	1	583,33	35,2
36	3	1	583,33	50,5
37	3	1	291,66	45,9
38	3	1	583,33	48
39	3	1	291,66	47,7
40	3	1	583,33	31,7
1	3	2	0,00	21
2	3	2	583,33	34,3
3	3	2	583,33	24
4	3	2	583,33	43,7
5	3	2	583,33	19,6
6	3	2	583,33	31
7	3	2	450,00	46,4
8	3	2	466,67	44,7
9	3	2	583,33	32,9
10	3	2	583,33	43,6
11	3	2	583,33	26,6
12	3	2	583,33	36
13	3	2	583,33	28,4
14	3	2	583,33	52,7
15	3	2	583,33	35,9
16	3	2	583,33	57,6
17	3	2	500,00	32,6
18	3	2	666,67	43,3
19	3	2	583,33	35,7
20	3	2	291,66	37,1
21	3	2	583,33	26,6
22	3	2	583,33	45,3
23	3	2	583,33	30,4
24	3	2	583,33	53
25	3	2	583,33	34,4
26	3	2	583,33	57,9
27	3	2	583,33	40,6
28	3	2	583,33	41,1

29	3	2	583,33	39
30	3	2	583,33	39,4
31	3	2	291,66	35,3
32	3	2	583,33	54,9
33	3	2	291,66	36
34	3	2	583,33	33,3
35	3	2	583,33	35,2
36	3	2	583,33	50,5
37	3	2	291,66	45,9
38	3	2	583,33	48
39	3	2	291,66	47,7
40	3	2	450,00	38,9
1	4	1	583,33	27,3
2	4	1	0,00	21
3	4	1	0,00	24,8
4	4	1	583,33	29,7
5	4	1	583,33	30,4
6	4	1	583,33	31
7	4	1	583,33	44,9
8	4	1	583,33	39
9	4	1	0,00	41,4
10	4	1	583,33	28
11	4	1	583,33	26,3
12	4	1	291,66	31,9
13	4	1	583,33	25
14	4	1	583,33	27,1
15	4	1	583,33	27,1
16	4	1	583,33	34,7
17	4	1	0,00	8,7
18	4	1	583,33	46,9
19	4	1	583,33	31,2
20	4	1	583,33	33,8
21	4	1	583,33	22,6
22	4	1	583,33	28,8
23	4	1	583,33	31,5
24	4	1	291,66	29,6
25	4	1	0,00	24,6
26	4	1	583,33	45,8
27	4	1	0,00	18,9
28	4	1	0,00	28,7
29	4	1	583,33	30
30	4	1	583,33	19,4
31	4	1	291,66	34,8
32	4	1	583,33	45
33	4	1	583,33	27,2
34	4	1	583,33	26,1

35	4	1	583,33	26,2
36	4	1	583,33	43,6
37	4	1	583,33	41,1
38	4	1	583,33	31,4
39	4	1	0,00	36,7
40	4	1	583,33	27,4
1	4	2	583,33	27,3
2	4	2	0,00	21
3	4	2	0,00	24,8
4	4	2	583,33	29,7
5	4	2	583,33	30,4
6	4	2	583,33	31
7	4	2	583,33	53,8
8	4	2	0,00	39
9	4	2	0,00	43
10	4	2	583,33	28
11	4	2	583,33	26,3
12	4	2	291,66	31,9
13	4	2	583,33	29
14	4	2	633,33	23,6
15	4	2	583,33	27,1
16	4	2	583,33	34,7
17	4	2	666,67	20,9
18	4	2	583,33	46,9
19	4	2	583,33	31,2
20	4	2	583,33	33,8
21	4	2	666,67	21,1
22	4	2	583,33	28,8
23	4	2	583,33	31,5
24	4	2	291,66	29,6
25	4	2	0,00	24,6
26	4	2	583,33	45,8
27	4	2	0,00	18,9
28	4	2	0,00	28,7
29	4	2	583,33	30
30	4	2	666,67	25,9
31	4	2	291,66	34,8
32	4	2	583,33	45
33	4	2	583,33	27,2
34	4	2	583,33	26,1
35	4	2	583,33	26,2
36	4	2	583,33	43,6
37	4	2	583,33	41,1
38	4	2	583,33	31,4
39	4	2	0,00	36,7
40	4	2	583,33	27,4

1	5	1	583,33	26,7
2	5	1	583,33	34,1
3	5	1	0,00	22,3
4	5	1	583,33	21
5	5	1	583,33	20,3
6	5	1	583,33	28,9
7	5	1	583,33	35,2
8	5	1	583,33	38,8
9	5	1	583,33	29,7
10	5	1	583,33	38,3
11	5	1	291,66	24,9
12	5	1	145,83	27,8
13	5	1	583,33	31,6
14	5	1	583,33	25,4
15	5	1	583,33	20
16	5	1	583,33	35,7
17	5	1	583,33	31,3
18	5	1	583,33	33,3
19	5	1	583,33	34,5
20	5	1	583,33	20
21	5	1	583,33	23,8
22	5	1	583,33	30,4
23	5	1	0,00	29,5
24	5	1	583,33	18,9
25	5	1	583,33	32,6
26	5	1	583,33	28,7
27	5	1	583,33	15,6
28	5	1	583,33	32,5
29	5	1	583,33	30,3
30	5	1	583,33	16,5
31	5	1	583,33	25,1
32	5	1	583,33	31,3
33	5	1	583,33	30
34	5	1	583,33	31,2
35	5	1	583,33	10,1
36	5	1	583,33	24,8
37	5	1	291,66	17,8
38	5	1	583,33	26,3
39	5	1	583,33	32,4
40	5	1	583,33	22,3

			5 year	
**ID**	**Loupe**	**Distance**	**Visual Acuity**	**Neck angulation**
1	1	1	291,66	19
2	1	1	0,00	29,7
3	1	1	583,33	41,4
4	1	1	583,33	22,4
5	1	1	145,83	17,3
6	1	1	0,00	46,9
7	1	1	583,33	24,7
8	1	1	291,66	40
9	1	1	291,66	35,7
10	1	1	291,66	46
11	1	1	145,83	42,5
12	1	1	291,66	42,7
13	1	1	291,66	34,4
14	1	1	291,66	37,8
15	1	1	291,66	36
16	1	1	0,00	38
17	1	1	145,83	23,9
18	1	1	291,66	38
19	1	1	145,83	39,5
20	1	1	145,83	30,2
21	1	1	0,00	33,1
22	1	1	145,83	31,6
23	1	1	291,66	48,1
24	1	1	291,66	41,4
25	1	1	145,83	24,5
26	1	1	291,66	34,7
27	1	1	0,00	35,4
28	1	1	583,33	56,8
29	1	1	0,00	34,1
30	1	1	0,00	36,2
31	1	1	583,33	44
32	1	1	583,33	47,3
33	1	1	0,00	37,3
34	1	1	0,00	30,7
35	1	1	145,83	38,3
36	1	1	0,00	41
37	1	1	291,66	30
38	1	1	583,33	44,5
39	1	1	145,83	36,4
40	1	1	0,00	46,7
1	1	2	183,33	22,4
2	1	2	0,00	47,1
3	1	2	583,33	41,4
4	1	2	0,00	39,2

5	1	2	333,33	62,2
6	1	2	0,00	63,7
7	1	2	583,33	24,7
8	1	2	191,67	66,1
9	1	2	416,67	53,9
10	1	2	500,00	61,9
11	1	2	350,00	69,6
12	1	2	175,00	60,6
13	1	2	225,00	34,4
14	1	2	200,00	78,4
15	1	2	291,66	56,2
16	1	2	216,67	75,8
17	1	2	383,33	65,3
18	1	2	350,00	77,4
19	1	2	350,00	66,7
20	1	2	450,00	51,1
21	1	2	466,67	57,7
22	1	2	466,67	68,6
23	1	2	233,33	75,5
24	1	2	450,00	62,4
25	1	2	112,50	57,5
26	1	2	466,67	47,9
27	1	2	87,50	54
28	1	2	333,33	63
29	1	2	0,00	34,1
30	1	2	466,67	55,2
31	1	2	350,00	63,7
32	1	2	466,67	57,6
33	1	2	0,00	41,9
34	1	2	500,00	63,2
35	1	2	466,67	51,9
36	1	2	0,00	67,3
37	1	2	216,67	71
38	1	2	40,00	54,1
39	1	2	483,33	49,8
40	1	2	0,00	46
1	2	1	0,00	24,4
2	2	1	0,00	32,2
3	2	1	145,83	30
4	2	1	0,00	50,7
5	2	1	0,00	26,4
6	2	1	0,00	31
7	2	1	0,00	26,6
8	2	1	0,00	44,5
9	2	1	0,00	50,8
10	2	1	0,00	41

11	2	1	0,00	32,9
12	2	1	0,00	47,7
13	2	1	0,00	36,3
14	2	1	0,00	34,6
15	2	1	0,00	36,7
16	2	1	0,00	25,1
17	2	1	0,00	44,4
18	2	1	0,00	28,2
19	2	1	0,00	23,7
20	2	1	0,00	29,1
21	2	1	0,00	38,2
22	2	1	0,00	27,5
23	2	1	0,00	55,7
24	2	1	0,00	42,8
25	2	1	0,00	36,7
26	2	1	0,00	29,4
27	2	1	0,00	44,6
28	2	1	0,00	42,2
29	2	1	0,00	32,3
30	2	1	0,00	38,3
31	2	1	0,00	37,3
32	2	1	0,00	57,6
33	2	1	145,83	35,1
34	2	1	0,00	21,9
35	2	1	0,00	24,8
36	2	1	0,00	44,5
37	2	1	145,83	24,4
38	2	1	0,00	27
39	2	1	0,00	39,6
40	2	1	0,00	28,3
1	2	2	0,00	49,7
2	2	2	350,00	51,7
3	2	2	333,33	56,7
4	2	2	166,67	57,7
5	2	2	383,33	55,4
6	2	2	150,00	36,4
7	2	2	225,00	40,2
8	2	2	233,33	67,5
9	2	2	450,00	58,5
10	2	2	450,00	61,3
11	2	2	283,33	67,1
12	2	2	283,33	61,6
13	2	2	350,00	36,3
14	2	2	350,00	68
15	2	2	350,00	66
16	2	2	0,00	52

17	2	2	350,00	67
18	2	2	350,00	46,8
19	2	2	87,50	67,4
20	2	2	0,00	47,9
21	2	2	87,50	72,2
22	2	2	175,00	62,1
23	2	2	283,33	74,7
24	2	2	433,33	66,8
25	2	2	175,00	51,9
26	2	2	283,33	73,7
27	2	2	150,00	64,1
28	2	2	283,33	64,3
29	2	2	416,67	47,4
30	2	2	283,33	59,3
31	2	2	283,33	64
32	2	2	300,00	38,3
33	2	2	450,00	39,8
34	2	2	333,33	67,3
35	2	2	333,33	53,1
36	2	2	0,00	61,3
37	2	2	500,00	41,4
38	2	2	400,00	52
39	2	2	416,67	52,7
40	2	2	225,00	41,9
1	3	1	583,33	36,4
2	3	1	583,33	40
3	3	1	583,33	48,7
4	3	1	583,33	36,5
5	3	1	291,66	17
6	3	1	583,33	27,8
7	3	1	583,33	35,9
8	3	1	583,33	35,9
9	3	1	583,33	30,5
10	3	1	583,33	29,7
11	3	1	583,33	32,4
12	3	1	583,33	42,6
13	3	1	583,33	54,4
14	3	1	583,33	35,4
15	3	1	583,33	35,7
16	3	1	583,33	35,8
17	3	1	583,33	44,8
18	3	1	583,33	45
19	3	1	583,33	38,1
20	3	1	291,66	26,6
21	3	1	291,66	42,5
22	3	1	583,33	25,3

23	3	1	583,33	41,1
24	3	1	583,33	43
25	3	1	583,33	26,6
26	3	1	583,33	41,7
27	3	1	291,66	45,7
28	3	1	291,66	50,5
29	3	1	291,66	31,1
30	3	1	583,33	43,7
31	3	1	583,33	45
32	3	1	291,66	53
33	3	1	583,33	32,7
34	3	1	583,33	52
35	3	1	0,00	38,6
36	3	1	0,00	38,9
37	3	1	583,33	30,6
38	3	1	583,33	19,4
39	3	1	583,33	34,5
40	3	1	583,33	37,1
1	3	2	583,33	36,4
2	3	2	583,33	40
3	3	2	533,33	48,7
4	3	2	583,33	36,5
5	3	2	291,66	17
6	3	2	583,33	27,8
7	3	2	583,33	35,9
8	3	2	483,33	46,5
9	3	2	583,33	30,5
10	3	2	583,33	29,7
11	3	2	583,33	32,4
12	3	2	583,33	42,6
13	3	2	583,33	54,4
14	3	2	550,00	35,4
15	3	2	583,33	35,7
16	3	2	583,33	47,1
17	3	2	583,33	44,8
18	3	2	583,33	45
19	3	2	583,33	38,1
20	3	2	291,66	26,6
21	3	2	350,00	42,5
22	3	2	583,33	25,3
23	3	2	583,33	41,1
24	3	2	583,33	43
25	3	2	583,33	73,3
26	3	2	583,33	41,7
27	3	2	291,66	45,7
28	3	2	291,66	50,5

29	3	2	291,66	31,1
30	3	2	583,33	43,7
31	3	2	583,33	45
32	3	2	291,66	53
33	3	2	583,33	32,7
34	3	2	583,33	52
35	3	2	0,00	38,6
36	3	2	0,00	38,9
37	3	2	583,33	33,5
38	3	2	583,33	19,4
39	3	2	500,00	49,5
40	3	2	583,33	37,1
1	4	1	583,33	34
2	4	1	583,33	30
3	4	1	583,33	36,7
4	4	1	583,33	29,9
5	4	1	583,33	23,2
6	4	1	583,33	21,4
7	4	1	583,33	27,8
8	4	1	145,83	40,4
9	4	1	583,33	32,7
10	4	1	291,66	35,9
11	4	1	583,33	42
12	4	1	583,33	40,8
13	4	1	583,33	50,2
14	4	1	583,33	31,6
15	4	1	583,33	45
16	4	1	583,33	35,1
17	4	1	583,33	35,5
18	4	1	583,33	45,6
19	4	1	583,33	29,9
20	4	1	583,33	24,7
21	4	1	291,66	32,1
22	4	1	291,66	37,7
23	4	1	583,33	35,2
24	4	1	583,33	37,9
25	4	1	583,33	34,2
26	4	1	583,33	31,3
27	4	1	583,33	30,4
28	4	1	583,33	46,3
29	4	1	583,33	33,5
30	4	1	583,33	39,1
31	4	1	583,33	37,9
32	4	1	583,33	47,9
33	4	1	583,33	33,9
34	4	1	583,33	27,6

35	4	1	583,33	34,8
36	4	1	583,33	37,3
37	4	1	291,66	27,3
38	4	1	583,33	34,3
39	4	1	583,33	40,6
40	4	1	583,33	23,9
1	4	2	583,33	34
2	4	2	666,67	30
3	4	2	583,33	36,7
4	4	2	583,33	29,9
5	4	2	583,33	23,2
6	4	2	583,33	21,4
7	4	2	583,33	27,8
8	4	2	145,83	40,4
9	4	2	583,33	32,7
10	4	2	291,66	35,9
11	4	2	583,33	42
12	4	2	583,33	40,8
13	4	2	583,33	50,2
14	4	2	583,33	31,6
15	4	2	583,33	45
16	4	2	583,33	35,1
17	4	2	583,33	35,5
18	4	2	583,33	45,6
19	4	2	583,33	29,9
20	4	2	583,33	24,7
21	4	2	291,66	43,7
22	4	2	291,66	37,7
23	4	2	583,33	35,2
24	4	2	583,33	37,9
25	4	2	583,33	34,2
26	4	2	583,33	31,3
27	4	2	583,33	30,4
28	4	2	583,33	46,3
29	4	2	583,33	33,5
30	4	2	583,33	39,1
31	4	2	583,33	37,9
32	4	2	583,33	47,9
33	4	2	583,33	33,9
34	4	2	583,33	27,6
35	4	2	583,33	34,8
36	4	2	583,33	37,3
37	4	2	291,66	27,3
38	4	2	583,33	34,3
39	4	2	583,33	40,6
40	4	2	583,33	23,9

1	5	1	583,33	15
2	5	1	583,33	26,3
3	5	1	583,33	25,7
4	5	1	583,33	33,6
5	5	1	583,33	24
6	5	1	583,33	18,6
7	5	1	583,33	25,4
8	5	1	583,33	31,2
9	5	1	583,33	20,7
10	5	1	583,33	24,8
11	5	1	583,33	27,3
12	5	1	583,33	24,8
13	5	1	583,33	17,7
14	5	1	583,33	34,5
15	5	1	583,33	21,2
16	5	1	583,33	16
17	5	1	583,33	19,8
18	5	1	583,33	23,6
19	5	1	583,33	13,4
20	5	1	583,33	14,5
21	5	1	583,33	32,1
22	5	1	583,33	26,6
23	5	1	583,33	33,5
24	5	1	583,33	34,8
25	5	1	583,33	39,3
26	5	1	583,33	12,5
27	5	1	583,33	21,3
28	5	1	583,33	20,9
29	5	1	583,33	25,3
30	5	1	583,33	22,6
31	5	1	583,33	18,9
32	5	1	583,33	23,5
33	5	1	583,33	18,8
34	5	1	583,33	36,4
35	5	1	583,33	29,2
36	5	1	583,33	30,6
37	5	1	583,33	16,9
38	5	1	583,33	17,2
39	5	1	583,33	23,8
40	5	1	583,33	25
